# Application of International Society for the Study of Women’s Sexual Health consensus algorithm for persistent genital arousal disorder/genito-pelvic dysesthesia to 10 cases and use of epidural spinal injections as long term management

**DOI:** 10.1093/sexmed/qfaf008

**Published:** 2025-03-02

**Authors:** Hannah Ahrendt, Salim Hayek, Sheryl Kingsberg, Anna Myers, Rachel Pope

**Affiliations:** Case Western Reserve University School of Medicine, Cleveland, OH, United States; Urology Institute, University Hospitals Cleveland Medical Center, Cleveland, OH, United States; Division of Pain Medicine, University Hospitals Cleveland Medical Center, Cleveland, OH, United States; Department of Reproductive Biology, Case Western Reserve University School of Medicine, Cleveland, OH, United States; Department of Psychology, Case Western Reserve University School of Medicine, Cleveland, OH, United States; Urology Institute, University Hospitals Cleveland Medical Center, Cleveland, OH, United States; Urology Institute, University Hospitals Cleveland Medical Center, Cleveland, OH, United States; Department of Reproductive Biology, Case Western Reserve University School of Medicine, Cleveland, OH, United States

**Keywords:** female sexual dysfunction, persistent genital arousal disorder, genito-pelvic dysesthesia, case series

## Abstract

**Introduction:**

Persistent genital arousal disorder/genito-pelvic dysesthesia (PGAD/GPD) is a debilitating, but poorly understood disorder. To address the lack of knowledge regarding mechanism and treatments, the International Society for the Study of Women’s Sexual Health (ISSWSH) consensus statement proposed a region-based approach for management of PGAD/GPD, including possible etiologies. Annular tears of the lumbar intervertebral disc are a recently acknowledged etiology of PGAD/GPD, and current evidence suggests that management of symptomatic tears resistant to non-invasive treatment may require lumbar endoscopic spinal surgery.

**Aim:**

This case series offers 10 cases of PGAD/GPD symptoms, in order to describe resource efficient management, including use of epidural spinal injections to reduce barriers to care for this debilitating condition.

**Methods:**

Individuals were identified by investigators in clinical practice. Electronic medical record notes and relevant imaging from the past 3 years were reviewed.

**Results:**

Half of the patients tried three or more treatments before finding any symptomatic relief. Two patients, with annular tears evident on magnetic resonance imaging (MRI), found complete relief with epidural spinal injections. A patient with hypertonic pelvic floor found total relief with pelvic floor physical therapy. Two patients found alleviation of symptoms with discontinuation of triggering medications, and four patients had palliation of symptoms with gabapentin and/or pregabalin.

**Conclusion:**

These cases demonstrate the utility of the ISSWSH consensus algorithm in guiding initial diagnosis and treatment of PGAD/GPD. However, flexibility is important in management to choose the appropriate treatment pathway to provide the most effective symptom management. Current evidence suggests the use of epidural spinal injections for temporary symptom relief, however, this case series suggests its use for long term management.

## Introduction

Persistent genital arousal disorder/genito-pelvic dysesthesia (PGAD/GPD) is a debilitating but poorly understood condition without formal diagnostic criteria. A recent International Society for the Study of Women’s Sexual Health (ISSWSH) consensus statement delineated diagnosis of PGAD/GPD as “persistent or recurrent, unwanted or distressing feelings of genital arousal or genito-pelvic dysesthesia, in absence of sexual interest or arousal, for at least 3 months.”^1^^,^^2^ The estimated global prevalence ranges from 0.6% to 3%, due to the lack of formal diagnostic criteria, small sample sizes and shame surrounding the condition, preventing disclosure.^1-3^

Barriers to care and difficulties in diagnosing and treating PGAD/GPD can cause significant negative physical and psychological consequences for patients. In one study, the majority of patients reported significant impacts of symptoms on day to day activities and up to 54% of patients reported suicidal ideation.^4^

**Figure 1:**
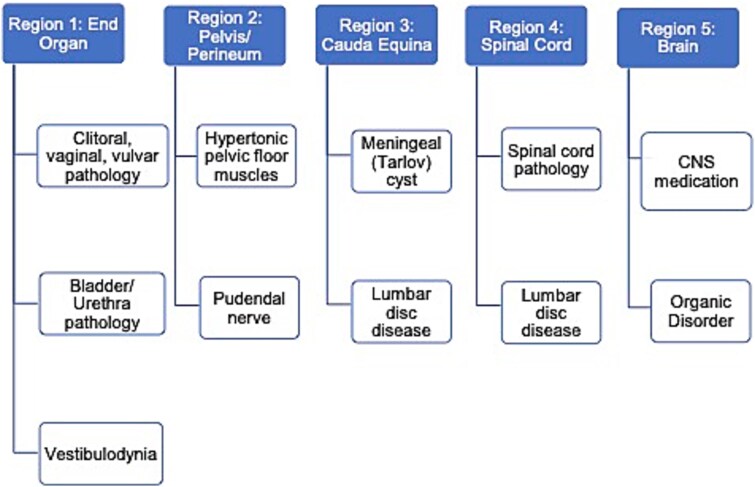
Etiological regions proposed by ISSWSH consensus algorithm, adapted from Goldstein, et al. ISSWSH: International Society for the Study of Women’s Sexual Health

The multifactorial nature of PGAD/GPD poses challenges to diagnosis and treatment.^1^^,^^5^ The ISSWSH consensus statement proposed an algorithm for management of PGAD/GPD.^2^ This algorithm divides potential PGAD/GPD etiologies into 5 regions: end organ, pelvis/perineum, cauda equina, spinal cord, and brain (Figure 1).^2^ Systematic physical examination can guide management, starting with topical anesthesia and pudendal nerve block to evaluate end organ and pelvic region involvement, respectively. If resources are available, providers can consider further diagnostic testing with quantitative genital sensory testing and magnetic resonance imaging (MRI).^2^^,^^6^

Lumbar/lumbosacral intervertebral disc annular tears have previously been identified as causes of radiculopathy and PGAD/GPD symptoms.^6^ Annular tears are described as a defect in the annulus fibrosis associated with inflammatory changes, which can lead to discogenic pain, disc displacement and radicular pain; but have also been associated with PGAD/GPD. Transforaminal epidural steroid injection (TFESI) to confirm pathogenesis, followed by lumbar endoscopic spinal surgery (LESS) has previously been described as a treatment option for PGAD/GPD symptoms secondary to lumbar annular tear pathology.^6^ The authors in that study reported only transient PGAD/GPD symptom relief with the TFESI (though some patients received local anesthetics only without steroids for diagnostic purposes), and suggested LESS as a long term solution for PGAD/GPD symptom relief.

The following case series illustrates management of PGAD/GPD and use of TFESI for long term symptom relief.

## Methods

Individuals were identified by investigators in clinical practice. IRB exemption was obtained from University Hospitals. Chart notes and relevant imaging in the past 3 years was reviewed for each individual.

### Case 1

JR is a 69-year-old female who presented with acute onset severe genital arousal, with which she coped through constant walking. JR reported arousal to the point of orgasm, but denied spontaneous orgasms. JR was taking mirtazapine on presentation, which was discontinued with subsequent major symptom resolution. Physical exam was unremarkable. MRI demonstrated degenerative lumbar changes with spondylosis at L4. She found temporary relief with oral pseudoephedrine. She received a TFESI, which provided 24 hours of relief. Ultimately, a combination of pregabalin, gabapentin, and psychotherapy managed JR’s symptoms.

### Case 2

TK is a 39-year-old female who presented with a 3-month history of unremitting genital arousal and up to twenty orgasms daily. Her PGAD/GPD symptoms began paradoxically immediately following transforaminal lumbar ESI with dexamethasone, which she had received prior without similar symptoms. She was tapering trazodone at time of presentation. TK has a history of intermittent dyspareunia and vulvar burning and itching. Overall, physical exam was unremarkable. MRI showed an annular tear at L4/5.

She found moderate relief with topical lidocaine and pregabalin, with an increased dose of duloxetine. Ultimately, TK received a bilateral L4/5 TFESI with 0.5% lidocaine and 20 mg methylprednisolone, which provided a few months of relief and was repeated for continued management without diminishing effects. In general, for TFESI, dexamethasone is attempted first as it is safer but if pain relief is short-lasting, methylprednisolone is attempted.^7^ TK also started amitriptyline, which may have contributed to relief. Throughout this process, TK followed with a psychologist to develop cognitive behavioral tools to help cope with her symptoms.

### Case 3

DK is a 63-year-old female who presented with four-year history of recurrent, unwanted and bothersome genital tingling and arousal, worsening with masturbation and unresolving with orgasm. Arousal began intermittently, but progressed to constant. DK endorsed depressive symptoms secondary to PGAD/GPD. DK has a history of hypertonic pelvic floor muscles. Physical exam was unremarkable except for enlarged clitoris. MRI demonstrated lumbar spondylosis and L4/5 annular tear. DK did not get relief from pudendal nerve block or pregabalin. DK found moderate symptomatic relief with vaginal suppositories, topical lidocaine, pelvic floor botox and pelvic floor physical therapy (PFPT). She received therapeutic TFESI with 0.5% lidocaine and 5 mg dexamethasone, which helped relieve her symptoms for 2 to 3 months but a similar repeat procedure did not help. She is planning further follow up and may be considered for a repeat procedure using methylprednisolone. DK found duloxetine and cognitive behavioral therapy with a psychologist experienced with PGAD/GPD helpful to manage suicidal ideation.

### Case 4

DB is a 64-year-old female who presented with a 6-month history of constant arousal, since starting pregabalin for lumbar radicular pain. Physical exam revealed normal external genitalia with excessive lubrication and hyperemic vaginal tissue. MRI revealed L3/4disc displacement with left sided septated facet joint cyst resulting in moderate central canal stenosis, L4/5 disc protrusion, likely impinging upon L4 nerve root and L5/S1 broad-based central disc protrusion, asymmetric in the left paracentral region. When pregabalin was replaced with topiramate for chronic pain, DB’s symptoms improved but didn’t resolve. DB found complete resolution of symptoms following diagnostic right L4/5 TFESI, with 2% lidocaine, followed by percutaneous disc decompression.^8^ Patient received an L5/S1 microdiscectomy a year and a half later, and has not reported recurrence of symptoms 6 months later.

### Case 5

EK is a 45-year-old female who presented with a three-year history of intermittent arousal and clitoral fullness without relief from orgasm. EK has a history of hypertonic pelvic floor muscles. On physical exam, she had mild anterior vaginal prolapse; hyperemic vestibule with a cyst on the left vestibule. EK reported resolution in her feeling of arousal with PFPT.

### Case 6

DG is a 77-year-old female who presented with a constant sensation of arousal since childhood. Physical exam was unremarkable. Her MRI showed lumbar degenerative changes with spinal stenosis at L3/4. DG discontinued trazodone and found lorazepam to be the only medication that provided any relief. She was evaluated by a pain medicine specialist and no spinal intervention was recommended.

### Case 7

EL is a 73-year-old female who presented with daily arousal for the past 3 years, worsening with movement and at night. She also experiences left sided lower back and leg pain, which begins with the arousal. Physical examination was unremarkable. MRI demonstrated severe lumbar foraminal stenosis. EL found moderate improvement with gabapentin three times daily and quetiapine before bed.

### Case 8

JC is a 73-year-old female who presented with constant genital arousal and multiple orgasms per day, with constant sacral pain, with a history of sacrospinous ligament fixation. MRI showed degenerative L4/5 changes. Vaginal suppositories, topical lidocaine and PFPT were unhelpful. Tamsulosin, for bladder emptying, bilateral pudendal nerve blocks, pelvic floor trigger point injections and duloxetine provided symptomatic relief. She found working with a psychologist knowledgeable about PGAD/GPD to be helpful in decreasing suicidal ideation secondary to symptoms.

### Case 9

ST is a 40-year-old female who presented with a multiple year history of significant superficial, burning vulvar and clitoral pain but no arousal. Her MRI demonstrated a Tarlov cyst near the S3 nerve root. Initial treatments included pelvic floor botox, topical estrogen/testosterone, which was moderately helpful, and later PFPT and gabapentin, which helped but did not resolve the pain. She was underwent a caudal epidural steroid injection with the needle advanced in the caudal canal to S4, injecting 0.5% lidocaine and 40 mg methylprednisolone, without improvement in her pain. Another gynecologist performed a hysterectomy, revealing endometriosis on the pelvic sidewall and posterior cul-de-sac, without improvement of vestibulodynia and clitorodynia.

### Case 10

EW is a 35-year-old female with a 12-year history of periodic internal burning and sharp pain in the vagina and clitoris, with arousal throughout the day. EW has a history of endometriosis, clitorodynia and pudendal neuralgia. Physical exam demonstrated right labia majora and posterior vaginal introitus tenderness with hypertonic pelvic floor muscles. MRI showed L5/S1 annular tear. Initially, EW’s symptoms were moderately controlled with topiramate, duloxetine and pelvic floor botox. EW had two caudal epidural steroid injections with 40 mg methylprednisolone that relieved her symptoms better than a bilateral L5/S1TFESI, with 5 mg dexamethasone, but with only 1-month duration of symptomatic relief following each caudal injection. She then pursued neurolysis of sciatic nerve bilaterally with some symptomatic improvement. Ultimately, she discontinued topiramate and managed symptoms with pregabalin and elagolix.

## Discussion

These cases demonstrate the broad spectrum of PGAD/GPD symptoms and options for symptom management. Although the ISSWSH algorithm provides a helpful, systematic manner for evaluating patients with PGAD/GPD, this is not always a realistic option in a resource limited setting or when patients see multiple providers simultaneously. For example, quantitative genital sensory testing and sometimes MRI, are resources that may not be readily available for patients. Therefore, this case series aims to present examples of resources efficient management of PGAD/GPD patients, with initial treatments aimed at clear triggers, if present. For example, in Case 2, JR presented with onset of symptoms after starting mirtazapine. The clear first step was to discontinue this medication, which resolved a majority of symptoms. In Case 7, EK presented with hypertonic pelvic floor muscles and found resolution of her symptoms with PFPT. Additionally, two patients in this series experience suicidal ideation secondary to PGAD/GPD symptoms. Therefore, immediate symptom relief was prioritized over invasive testing and imaging. However, only three of ten patients followed with a psychologist, highlighting an underutilized treatment aspect of PGAD/GPD. Overall, the ISSWSH algorithm^2^ provides a starting point for treatment, with the caveat that providers likely need to pursue more than one pathway simultaneously for adequate symptom control.

This case series highlights the use of TFESI as a minimally invasive approach to alleviate symptoms related to PGAD/GPD, in the setting of annular tears. Due to the multifactorial nature of PGAD/GPD, all patients received one or more treatments in addition to TFESI. Although it’s challenging to determine the annular tear as a direct cause of the PGAD/GPD symptoms, these cases support evidence for the role of annular tears in PGAD/GPD symptoms as previously presented by Kim et al.^6^ Two patients in this series had complete resolution of symptoms with TFESI and another had one month of symptom relief. Therefore, TFESI is a valid, lower cost, more accessible alternative to LESS. If symptoms continue to recur, such as in Cases 3 and 9, patients can continue to receive TFESI or be offered the option of spinal surgery, if that is viable. Of note, Case 3 is the first reported case of PGAD/GPD symptoms triggered by interlaminar ESI, then resolved by TFESI. For patients with suspected spinal etiologies, we suggest referral to an interventional pain medicine provider knowledgeable about PGAD/GPD, which helped a majority of PGAD patients with lumbar spine pathology find symptomatic relief.

These cases demonstrate the physical and mental toll PGAD/GPD can take on patients. All four patients who saw a mental health professional for support, found it helpful in coping. This highlights an important, but vastly underutilized resource in the care of patients with PGAD/GPD.

We acknowledge the significant limitations of this case series, including the limited size and retrospective nature, limit generalizability of findings. Treatment options presented in this case series are largely experimental, as there is minimal data regarding treatments for PGAD/GPD. This is a rapidly evolving area of research, so this work is still in progress. Future directions of research include prospective studies evaluating long term efficacy of TFESI in patients with annular tears and PGAD/GPD symptoms, as well as consistency of efficacy of repeated injections.

## Conclusion

This series demonstrates the complex, multifactorial nature of PGAD/GPD, with a resource efficient application of the ISSWSH consensus algorithm These cases demonstrate the use of TFESI as an effective long term management option for patients with PGAD/GPD symptoms and annular tears. This series illustrates the importance of involving pain medicine specialists and psychologists in patient management.
